# The 2025 FELASA Congress in Athens: From Aristotle to AI

**DOI:** 10.1038/s41684-025-01651-z

**Published:** 2025-11-28

**Authors:** Nuno Henrique Franco, Otto Kalliokoski, Vootele Voikar

**Affiliations:** 1 I3S – Instituto de Investigação e Inovação em Saúde, Universidade do Porto, Porto, Portugal; 2 Department of Veterinary and Animal Sciences, University of Copenhagen, Copenhagen, Denmark; 3 Laboratory Animal Center, Helsinki Institute of Life Science, University of Helsinki, Helsinki, Finland

**Keywords:** Scientific community, Zoology

## Abstract

The 16^th^ FELASA Congress took place June 2025 in the cradle of science, Athens. Upon arrival, more than 2,000 professionals from 52 countries were welcomed by “Aristotle the Octopus”, a mascot alluding to Greek scientific heritage, Aristotle’s pioneering zoological observations and the values of intelligence, adaptability and ethics, which are central to laboratory animal science (LAS). The vibrant and friendly atmosphere was fertile ground for discussing advancements in LAS, regulation, education and ethics, but also for catching up with friends and colleagues, exchange ideas and start new collaborations. Having been involved in many aspects of the meeting – chairing sessions, delivering talks and workshops – we will share some insights and personal reflections on the meeting.

**Figure:**
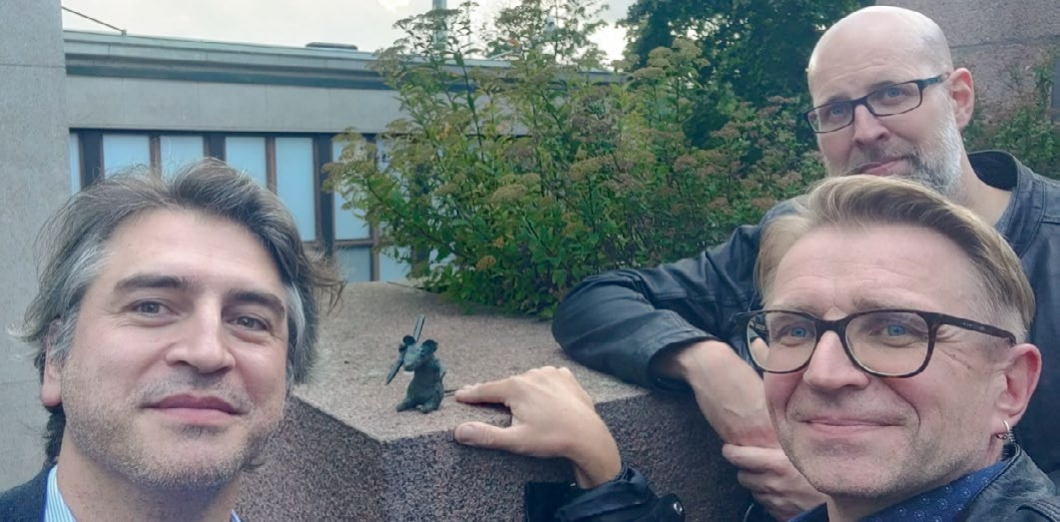
The authors and Wise Mouse (Viisas Hiiri, by artist Jyrki Siukonen) in Helsinki. Credit: Nuno Franco.

The authors and Wise Mouse (Viisas Hiiri, by artist Jyrki Siukonen) in Helsinki. Credit: Nuno Franco.

The congress celebrated the 30^th^ anniversary of the Hellenic Society of Biomedical and Laboratory Animal Science (HSBLAS), who emphatically proved that a small FELASA member association can also deliver a truly successful congress. The three-and-a-half days were filled with an immersive program. At any given time, it was hard to choose which of the six sessions running in parallel to attend. The congress ran for ten hours a day, totalling 63 scientific sessions and 34 workshops. In-between the main sessions, 361 electronic posters were presented across 40 sessions. Moreover, the congress was preceded by the first World Meeting for Laboratory Animal Veterinarians. This one-day event was organized by the International Association for the Colleges of Laboratory Animal Medicine (IACLAM), the European Society of Laboratory Animal Veterinarians (ESLAV) and FELASA, offering additional program for the FELASA meeting’s veterinary attendees. Add to this the usual sprinkling of satellite meetings and the odd connected summer school and it is fair to say that FELASA 2025 offered a comprehensive program and then some.

Each day was punctuated by an inspirational keynote talk. First out, Stasinos Stavrianeas (Hellenic Open University) explored “The Origins of Zoology and Aristotle’s Biology”, bridging classical philosophy and modern welfare thinking. “Is the Brain in the Goldilocks Zone?”, wondered George Paxinos (Scientia Professor at UNSW and Neuroscience Research Australia) in another keynote talk, drawing on his landmark neuroanatomical atlases while exploring welfare implications of brain science. Lastly, David Anderson (veterinary surgeon, former UK Home Office and currently EU advisor) received the 2025 FELASA Award for his substantial contributions with respect to animal welfare, before delivering his career retrospective “An Unexpected Journey – From Calving Cows to Championing Squid Welfare”, during which he merged regulatory history, hilarious personal anecdotes and species-inclusive ethics.

## Keeping up with recent developments in LAS

Each new FELASA congress gives us a chance to look back three years or more and reflect on what progress we made, and whether the goals set at the previous congress were met. For instance, the International Culture of Care Network, originally proposed by Thomas Bertelsen at the 2016 FELASA Congress in Brussels, now comprises around 60 individuals across 18 countries (norecopa.no/CoC). The newly established European Network of National Networks of Animal Welfare Bodies (ENAWB - norecopa.no/ENAWB), another brainchild of Bertelsen, proposed at the 2022 FELASA congress in Marseille, has already gained traction in 11 European countries. In Athens, the two networks had dedicated sessions where effective implementation of the Culture of Care globally was discussed, addressing matters such as cultural differences, availability of resources, institutional commitment and legal frameworks.

With the revision of the EU Education & Training Framework around the corner, several sessions and workshops covered this topic. The promised outcomes from EU-funded projects developed within, or made available by, the Education & Training Platform for Laboratory Animal Science (ETPLAS) were also shared during the congress: Ismene Dontas presented new assessment criteria for the EU function (b) (Designing Procedures and Projects)^1^; Nuno Franco’s rundown of the 23 e-learning modules (available at www.etplas.eu) emphasized that these online resources should be combined with face-to-face teaching, rather than just replacing it; Philippe Bugnon presented a new framework for mutual recognition of continuing professional development (CPD) across the EU. Also, under the auspices of ETPLAS, new harmonised DOPS (Direct Observation of Procedural Skills) sheets for competence assessment and how to apply them were presented by Rafael Frías. Rafael, together with Lucy Whitfield and Ivo Tiebosch, delivered talks and workshops that placed great emphasis on ensuring that those conducting experiments are adequately skilled and continuously evaluated. And indeed, as European Commission’s Susanna Louhimies pointed out, there are no excuses left for not developing expertise, given that achieving, assessing and maintaining competence are legal obligations, and thus non-negotiable.

While on the topic of teaching, and as trainers in experimental design, we would be amiss not to mention further aspects of this topic that were included in the FELASA congress. Once learners of our courses and workshops return to their respective laboratories, we are sometime saddened to find out that much of the knowledge we tried to impart has been forgotten or underused. Barriers include entrenched practices, resistance to change and pressure to publish “significant” results. We will also concede that ineffective teaching may be a contributing factor. Improving teaching of sound study design principles could contribute to improved replicability and translatability of animal studies. To address this issue, a FELASA working group has defined essential knowledge and skills needed for competent design, analysis and interpretation of animal experiments under Directive EU2010/63^2^. Nuno Franco, Manuel Berdoy and Derek Fry presented the recommendations of this working group and held a dedicated workshop on the topic.

## Reducing severity – an overarching theme

As expected, the main theme of the meeting, “Reducing severity in animal research”, was amply covered. Severity classification is required by law within the EU, but harmonisation and implementation thereof are still challenges on a national level. With that goal, prospective and actual severity classification workshops were held, led by David Anderson and Anne Dominique Degryse, who, for nearly a decade, have provided training not only to researchers, but also to trainers on the subject. For severity classification, Clinical Score Sheets are indispensable tools, listing specific symptoms and behaviours, while offering guidance on interventions with an overall aim of systematically assessing and documenting the welfare of laboratory animals. At FELASA 2025, the art of designing informative score sheets tailored to specific experimental needs was addressed in excellent talks by Dolores Bonaparte, Maike Heimann and Philippe Bugnon.

The “RSPCA – Animals in Science” team particularly highlighted their roadmap for ending severe procedures, which in the UK have dropped 64% since 2014, contrasting with the modest <2% drop since 2018 in the EU. It must be stressed that even if severe procedures are eliminated, any pain, discomfort or distress – however mild – must still be justified by the expected benefits, underscoring the need for a streamlined and practical approach to benefit assessment. In this regard, animal welfare bodies, ethics committees, competent authorities and scientists may differ not only in how to assess the ‘benefit’ of proposed research, but also in how they define the ‘benefit’ itself. To address this challenge, Penelope Reynolds presented the Benefit Assessment Matrix (BAM), a practical tool for specific evaluation of benefit that was designed to emphasise benefits associated with the research process, rather than criteria more commonly assessed by project evaluators. For a given research study, BAM would compute a Qualified Benefit score based on Proposed Benefit (research gap addressed, anticipated scientific impact, lack of nonanimal alternatives) weighted by Modifying Factors (design validity; technical capability). High scores suggest the proposed research has a good probability of producing scientific benefit in terms of meaningful and reliable information, whilst minimizing waste of animals in nonproductive research.

## New approaches to old problems

Not all themes need to be new to be topical. Much like experimental design, sex bias in preclinical research has been debated for over a decade, but it is a topic that continues to spark conversation. New frameworks still emerge – the most recent initiative, the Sex-Inclusive Research Framework (SIRF)^3^, was discussed at the congress, alongside ongoing efforts to integrate Sex as a Biological Variable (SABV) systematically into study design, which will be supported by a new COST Action (https://www.cost.eu/actions/CA24168/).

The existing problems and recommended solutions were convincingly summarized by Alexandra Bannach-Brown. Alexandra’s talk highlighted various tools and practices to facilitate the design and conduct of synthesis-ready research: preregistration, principles of open science and Findable, Accessible, Interoperable, and Reusable (FAIR) data, and methods for reporting animal research in a detailed and transparent manner^4^. The minimal metadata set (MNMS) to repurpose nonclinical in vivo data for biomedical research was also presented^5^.

Similarly, openness and transparency in communication and dissemination are critical elements in shaping the attitude towards, and acceptance of, animal research, with policymakers and the general public alike. EARA, the European Animal Research Association, was actively involved in several panels – advocating for transparency agreements and patient engagement.

## Great science in small formats

The meeting offered engaging communication in several forms. After its successful introduction in 2022, the 2025 congress revisited “My Oral Presentation in 180 Seconds”, a dynamic competition of short, impactful presentations submitted by technicians and researchers. An independent jury crowned Morten Malmberg (University of Copenhagen, Denmark) the winner for his speed-talk “Development of a climbing test in mice: a novel approach for assessing pain-like behaviour”, awarding him a free registration for the next FELASA Congress.

The same time constraint was given to the congress’ many poster presenters. Relaying the contents of one’s poster in only three minutes is a challenge that requires great presentation skills and command of the subject. Barbara Noronha Bastos (University of Porto, Portugal) received the scientific committee’s vote for the best poster (“We are just warming up: non-invasive monitoring of the poikilothermy-endothermy transition in mouse pups”), while the popular award was given to Diego Celdran (University of Tucson, USA) for poster presentation on “Multicenter validation study of a 3D printed mouse model for surgical training.” These numerous brief talks put the spotlight on early career researchers and technicians, the future and beating heart of the LAS community. The electronic poster screens were scattered across the congress venue; and even though the “silent disco” approach of projecting the speaker’s voice into headphones made available near the poster was a marked improvement over Marseille’s blaring speakers, being in the right place at the right time was challenging for the audience. A separate poster-viewing area was set up in the farthest corner of the basement floor for those that missed, or simply could not find, the poster presentation that they had hoped on attending. However, most attendees were relegated to peering at images of the posters on their phone screens, using the conference app. It is hard to not view this as a missed opportunity. Having chaired a number of these sessions, we were saddened to see that some of the most innovative scientific and technological developments – particularly those aimed at “reducing severity in animal research” (e.g. earlier humane endpoints, refined techniques, training and habituation protocols or innovations in housing and environmental enrichment) – were delivered mostly as short talks and posters by early-career researchers and technicians, despite deserving more time and a larger audience.

## The future – are we there yet?

A throughline for the meeting that could not be ignored was the incorporation of new technologies. Globally, we are amid an explosive adoption of machine learning/AI technology, which was clearly reflected by the scientific program. Considerable focus was given to home-cage monitoring technologies where machine learning has been a key driver. But there were also presentations more broadly showcasing the application of AI in the field of animal research. Automated systems and data-driven models are becoming central for collecting digital biomarkers, reducing subjectivity, increasing reproducibility and informing about animals’ physiology and behaviour, thereby aiding in timely detection of health and environmental disturbances. “The future is here”, a session boldly claimed. By contrast, most speakers steered clear of hyperbole as these are still early days, and these new technologies are best applied cautiously. Nonetheless, we were left wondering where these technologies will have taken us by the time we meet again at the 2027 FELASA Congress in Cologne, Germany, where again we will meet our colleagues and friends, and catch up on our advancements towards a more ethical, transparent and impactful animal research.

## References

[1] Dontas, I. A. et al. *Lab. Anim.* **58**, 626–639 (2024). 39308215 10.1177/00236772241244527 PMC11633073

[2] Fry, D. et al. *Lab. Anim.*10.1177/00236772241295308 (2025). 40485412 10.1177/00236772241295308 PMC12583620

[3] Karp, N. A. et al. *Nat. Commun.* **16**, 3763 (2025). 40263253 10.1038/s41467-025-58560-5 PMC12015461

[4] Janssens, M. et al. *Research Ideas and Outcomes* **9**, e105198 (2023).

[5] Moresis, A. et al. *Lab. Anim. (NY)* **53**, 67–79 (2024). 38438748 10.1038/s41684-024-01335-0 PMC10912024

